# Surgical Cavity Constriction and Local Progression Between Resection and Adjuvant Radiosurgery for Brain Metastases

**DOI:** 10.7759/cureus.575

**Published:** 2016-04-19

**Authors:** Jugal K Shah, Matthew B Potts, Penny K Sneed, Manish K Aghi, Michael W. McDermott

**Affiliations:** 1 Department of Neurosurgery, New York University Langone Medical Center; 2 Department of Neurological Surgery, Northwestern University Feinberg School of Medicine, Chicago, IL, USA; 3 Department of Radiation Oncology , University of California, San Francisco; 4 Department of Neurological Surgery, University of California, San Francisco

**Keywords:** resection cavity, brain metastasis, radio-surgery, radiosurgery, brain metastases, tumor resection

## Abstract

Stereotactic radiosurgery (SRS) to a surgical cavity after brain metastasis resection is a promising treatment for improving local control. The optimal timing of adjuvant SRS, however, has yet to be determined. Changes in resection cavity volume and local progression in the interval between surgery and SRS are likely important factors in deciding when to proceed with adjuvant SRS.

We conducted a retrospective review of patients with a brain metastasis treated with surgical resection followed by SRS to the resection cavity. Post-operative and pre-radiosurgery magnetic resonance imaging (MRI) was reviewed for evidence of cavity volume changes, amount of edema, and local tumor progression. Resection cavity volume and edema volume were measured using volumetric analysis.

We identified 21 consecutive patients with a brain metastasis treated with surgical resection and radiosurgery to the resection cavity. Mean age was 57 yrs. The most common site of metastasis was the frontal lobe (38%), and the most common primary neoplasms were lung adenocarcinoma and melanoma (24% each). The mean postoperative resection cavity volume was 7.8 cm^3^ and shrank to a mean of 4.5 cm^3^ at the time of repeat imaging for radiosurgical planning (median 41 days after initial post-operative MRI), resulting in a mean reduction in cavity volume of 43%. Patients who underwent pre-SRS imaging within 1 month of their initial post-operative MRI had a mean volume reduction of 13% compared to 61% in those whose pre-SRS imaging was ≥1 month (p=0.0003). Post-resection edema volume was not related to volume reduction (p=0.59). During the interval between MRIs, 52% of patients showed evidence of tumor progression within the resection cavity wall. There was no significant difference in local recurrence if the interval between resection and radiosurgery was <1 month (n=8) versus ≥1 month (n=13, p=0.46).

These data suggest that the surgical cavity after brain metastasis resection constricts over time with greater constriction seen in patients whose pre-SRS imaging is ≥1 month after initial post-operative imaging. Given that there was no difference in local recurrence rate, the data suggest there is benefit in waiting in order to treat a smaller resection cavity.

## Introduction

Metastases are the most common intracranial tumors in adults, affecting 10-40% of cancer patients and resulting in approximately 170,000 new cases per year [1-2]. Surgical resection followed by adjuvant whole brain radiation therapy (WBRT) has been the standard of care for treating brain metastases, with randomized controlled trials supporting its use [3]. However, WBRT is also associated with neurocognitive decline [4-5]. Recently, replacement of WBRT with adjuvant stereotactic radiosurgery (SRS) to the surgical resection cavity has been advocated as a means of providing local control while minimizing the adverse effects associated with WBRT, and several studies have demonstrated comparable control rates between SRS and WBRT [6]. Critical factors in the use of SRS as an adjuvant therapy after surgical resection cavities include prescription dosage [7], extent of treatment margins [8], and treated volume [7, 9]. Jarvis et al., have reported dynamic changes in brain metastasis resection cavity volume and showed that cavities may be just as likely to expand in the interval between surgery and SRS as they are to constrict [10]. Atalar et al., recently reported that the greatest amount of resection cavity constriction occurs in the immediate postoperative period (0-3 days and concluded that there is no benefit to waiting more than 1-2 weeks after resection to conduct SRS [11]. However, this group performed adjuvant SRS relatively soon after surgical resection (median time from surgery to SRS 20 days) with no patients undergoing a pre-SRS MRI scan more than 33 days after surgery. At our institution, timing of adjuvant SRS ranges from less than 2 weeks to more than 10 weeks after surgery. Therefore, we sought to determine if there were more long-term dynamic volume changes in the surgical resection cavity that would affect optimal timing of adjuvant SRS and whether there was any relationship to the amount of surrounding edema or the pathologic tumor type.

## Materials and methods

### Study population

This study was approved by the Institutional Review Board of the University of California, San Francisco, and conducted in compliance with the Health Insurance Portability and Accountability Act regulations. A prospective radiation oncology database was searched to identify patients who underwent adjuvant SRS after gross total resection of a brain metastasis at the University of California, San Francisco Medical Center between 1998 and 2009. Patients were excluded if they did not have both an immediate post-operative and a pre-SRS planning MRI available on which to perform volumetric analysis. We conducted a retrospective review of this database as well as medical records and post-operative and pre-SRS imaging. Recorded patient characteristics included age, gender, primary pathology, site of metastatic lesion, extent of surgical resection, and imaging characteristics as described below. Pre-SRS planning MRIs were obtained the same day that SRS was performed.

### Volumetric analysis of resection cavity

Volumetric analysis was performed on each patient’s immediate post-operative and pre-SRS planning MRIs. Briefly, freehand region-of-interest tracing of the resection cavity on precontrast T1 weighted images was performed on each slice through the cavity using iSite Enterprise 3.6.126.0 software (Koninklijke Philips Electronics N.V., Amsterdam, Netherlands). Total cavity volume was then calculated as the sum of the traced areas on each slice multiplied by the slice thickness. The volume of edema surrounding the resection cavity was similarly measured based on the region of increased signal intensity surrounding the resection cavity on fluid-attenuated inversion recovery (FLAIR) images. Total edema volume was then calculated by subtracting the cavity volume. In addition, the extent to which a resection cavity abutted dura was recorded by measuring the greatest length of abutment in the axial plane.

## Results

### Patient and treatment characteristics

Twenty-one patients were identified who underwent gross total resection of a single brain metastasis followed by adjuvant SRS at our institution between 1998 and 2009. Mean age at time of surgery was 57 years with 57% males. Table 1 details the baseline tumor locations and pathologies. The most common location was the frontal lobe (38%), and the most common primary pathologies were lung adenocarcinoma and melanoma (24% each).

**Table 1:** Baseline Patient Characteristics SD = standard deviation

Number	21
Age at surgery (mean ± SD)	56.5 ± 10.9y
Male	12 (57%)
Tumor location	
Frontal lobe	8 (38%)
Parietal lobe	4 (19%)
Occipital lobe	4 (19%)
Temporal lobe	3 (14%)
Cerebellum	2 (10%)
Pathology	
Lung	7 (33%)
Adenocarcinoma	5 (24%)
Small cell carcinoma	1 (5%)
Non-small cell carcinoma	1 (5%)
Melanoma	5 (24%)
Renal cell carcinoma	3 (14%)
Breast adenocarcinoma	1 (5%)
Colorectal adenocarcinoma	1 (5%)
Bladder urothelial carcinoma	1 (5%)
Ovarian carcinoma	1 (5%)
Testicular germ cell tumor	1 (5%)
Sarcoma	1 (5%)

### Characteristics of cavity volumes

**Figure 1:**
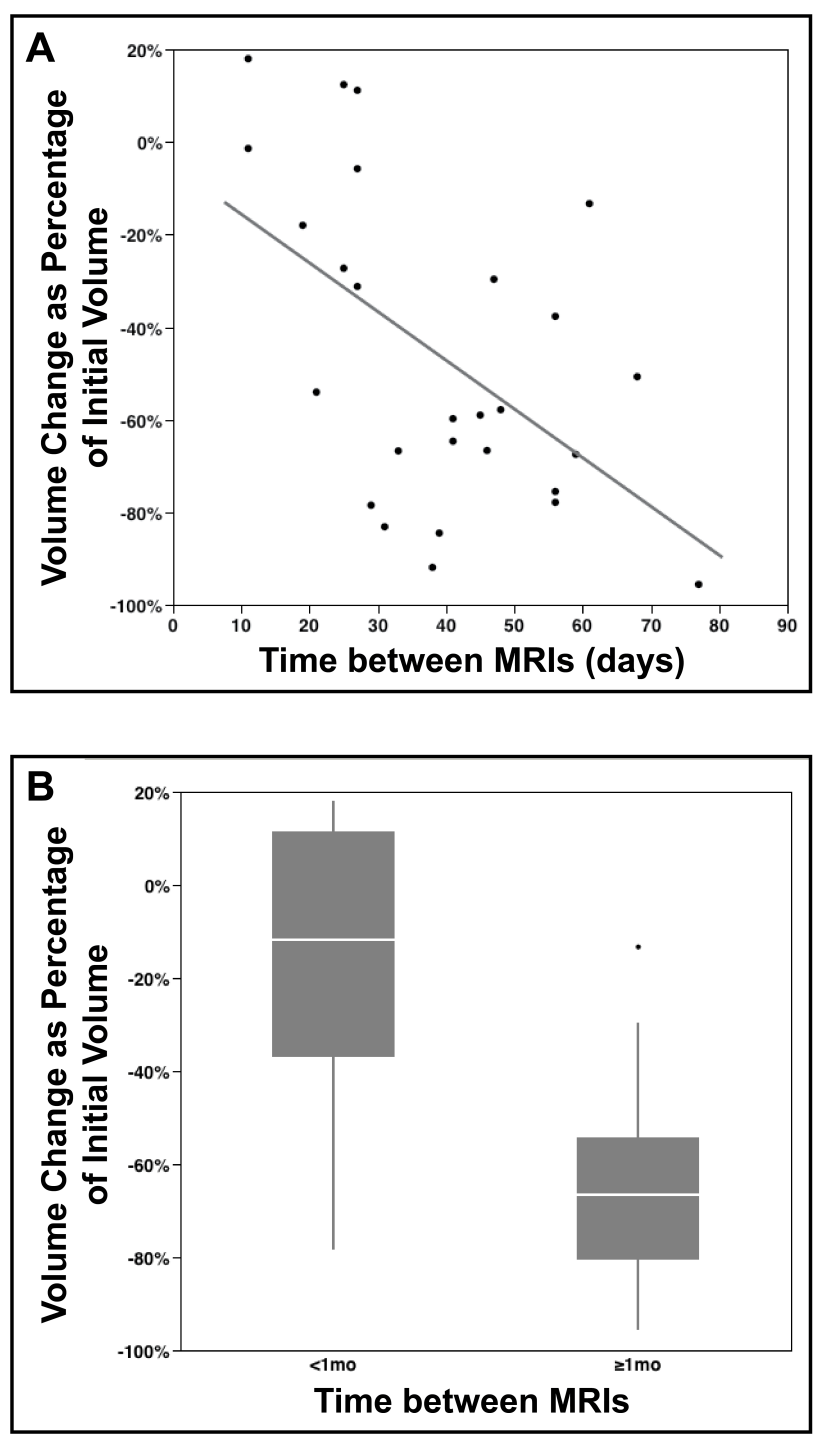
Resection cavity volume change versus time between initial postoperative and pre-SRS MRIs. (A) Scatter plot comparing the change in resection cavity volume versus time between initial postoperative and pre-SRS planning MRI. It can be seen that the two patients with cavity expansion had a pre-SRS MRI within 30 days of the initial postoperative scan. A best-fit line (R2=0.33) shows the general trend toward cavity volume constriction with time. (B) Box and whisker plot comparing the change in volume of patients whose MRI interval was <1 mo versus ≥1 mo. The mean between these two groups was significantly different (p=0.0003). The 1^st^ and 3^rd^ quartiles are represented by the lower and upper margins of the boxes, respectively, with the median represented by the white line within the boxes. The upper and lower vertical lines represent the highest and lowest data points, respectively, that fall within 1.5* interquartile range. Outliers beyond this range are represented by a dot.

**Figure 2:**
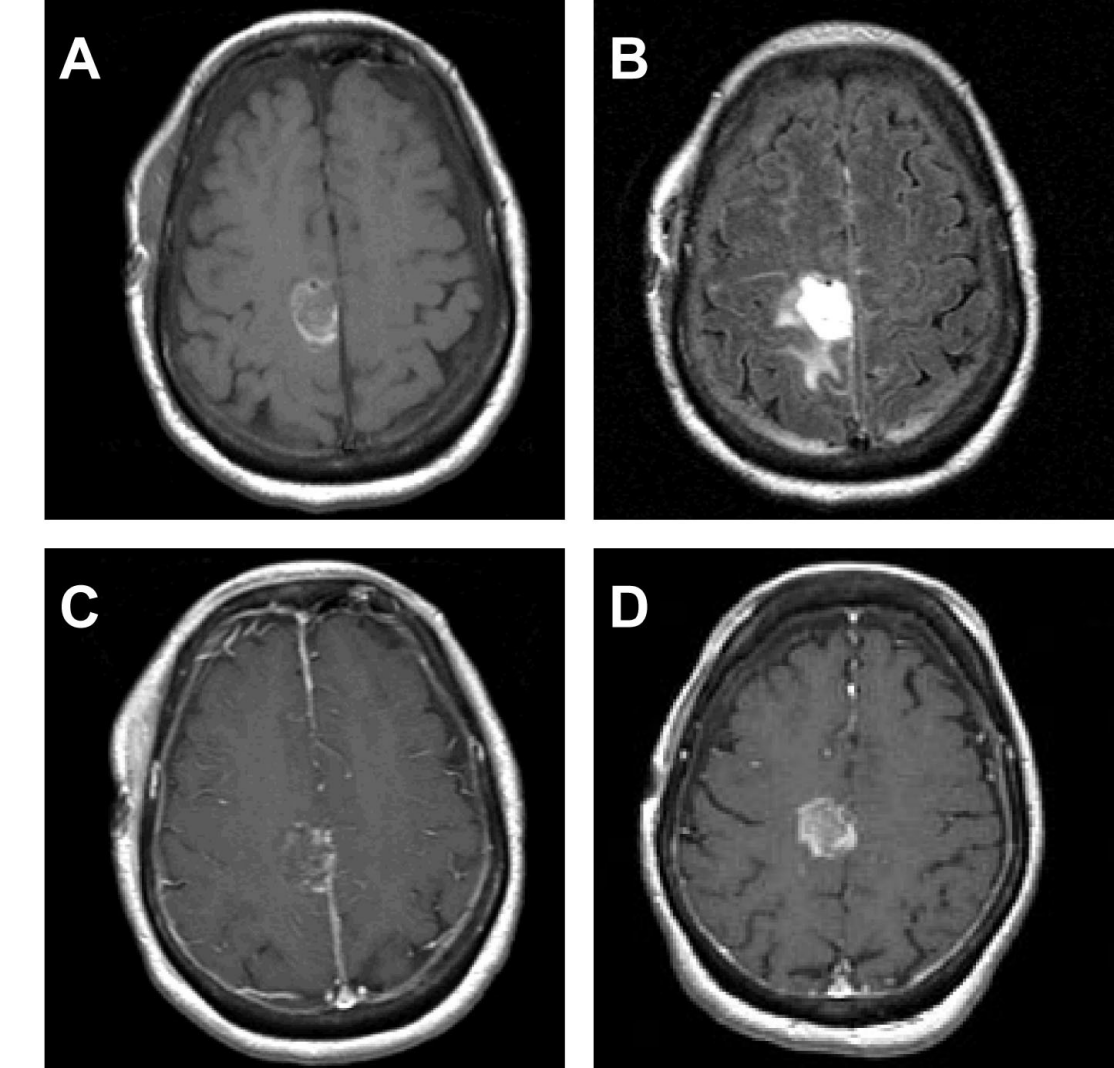
Case Example 1 This 62-year-old female underwent gross total resection of a right parietal small-cell lung carcinoma metastasis with planned adjuvant gamma knife radiosurgery. Her initial postoperative T1 weighted MRI (A) showed a resection cavity volume of 7.2 cm^3^ with 11.72 cm^3^ of surrounding edema on T2 weighted imaging (B). No enhancement was seen within the initial resection cavity (C). Her pre-SRS MRI was performed 19 days later and revealed a resection cavity of 5.9 cm^3^ (D), resulting in a volume reduction of 18%. The resection cavity also had evidence of local tumor progression on contrast-enhanced imaging (D).

**Figure 3:**
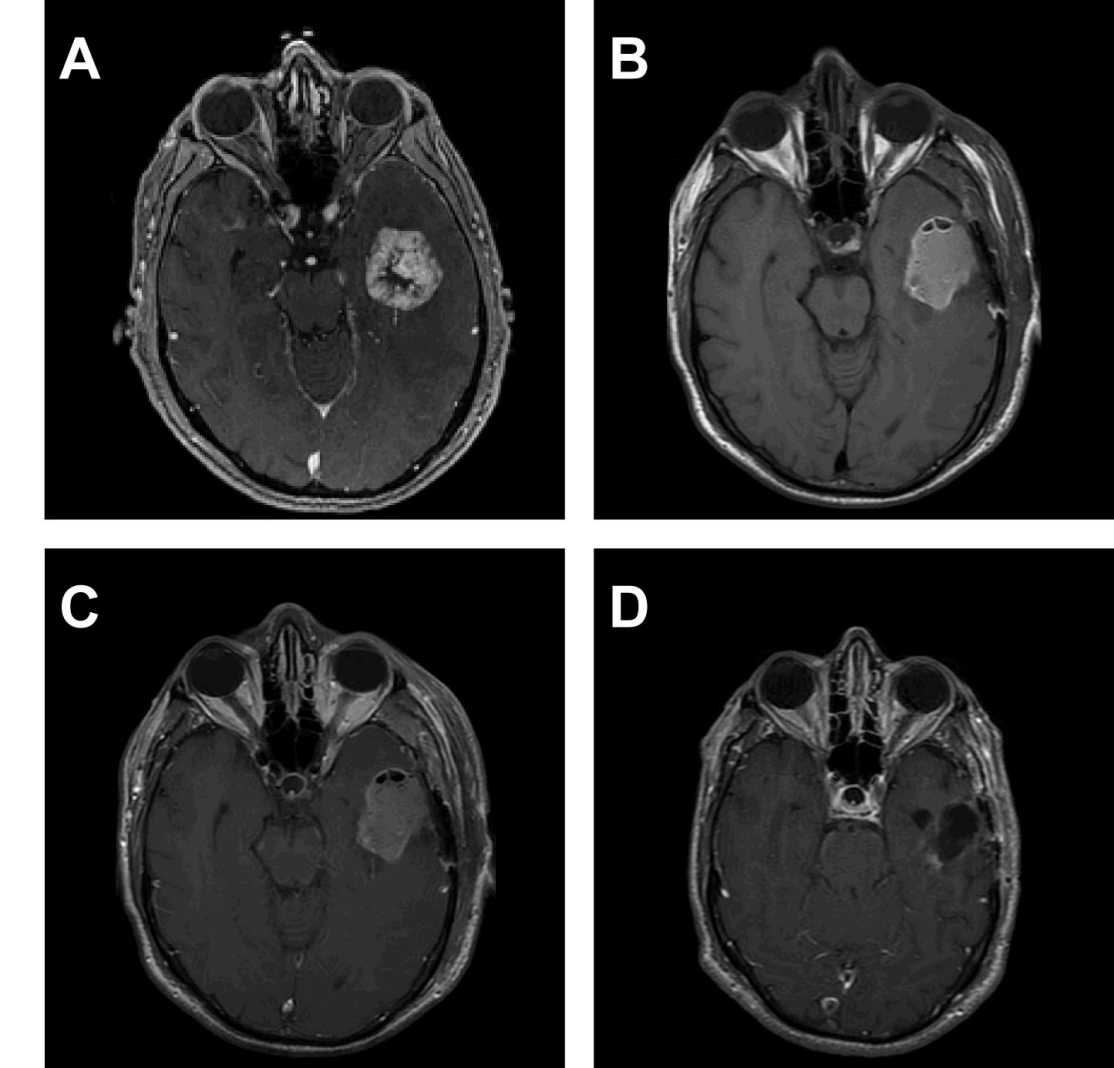
Case Example 2 This 58-year-old male underwent gross total resection of a left temporal renal cell carcinoma metastasis with planned Cyberknife radiosurgery. (A) Preoperative contrast enhanced T1 weighted MRI. Initial postoperative T1 weighted imaging (B) showed a largely blood filled resection cavity with a volume of 18.1 cm^3^. There was no residual enhancement seen on a contrast enhanced T1 weighted image. A pre-SRS planning MRI was performed 59 days later and revealed a resection cavity of 5.9 cm^3^ (67% volume reduction) with no evidence of tumor progression on contrast enhanced T1 weighted imaging (D).

All initial post-operative MRI studies were obtained within 2 days of surgery (median 2 days). Analysis of initial post-operative T1 weighted MRIs revealed a mean resection cavity volume of 7.8 cm^3^ (median 6.5 cm^3^, range 1.3-20.7 cm^3^). The median time to subsequent pre-SRS MRI was 39 days (range 11-77). Volumetric analysis of pre-SRS MRIs demonstrated a mean resection cavity size of 4.5 cm^3^ (median 3.3 cm^3^, range 0.3-23.3 cm^3^), with all but two patients showing cavity constriction. This resulted in an overall mean reduction in cavity volume of 3.3 cm^3^, corresponding to mean reduction of 43%. Figure 1a shows the relationship between change in cavity volume and time between the initial post-op and pre-SRS MRIs. Figure 1b compares volume change between patients with <1 month between MRIs and those with ≥1 month between MRIs, showing significantly greater constriction for patients with longer time between MRIs (mean 61% reduction in volume, Figure 2) compared to those with <1 month (13% reduction in volume; p=0.0003, t-test; Figure 3). Initial postoperative cavity volumes did not significantly differ between the <1 month and ≥1 month groups (mean 7.0 cm^3^ versus 8.2 cm^3^, respectively; p=0.63, t-test).

Further univariate analysis showed no significant relationship between percent change in cavity volume and surrounding edema (p=0.59, linear regression), presence of dural abutment (p=0.83, t-test), initial cavity volume (p=0.87, linear regression), and primary tumor type (overall, p=0.29).

Examination of the two patients whose resection cavities increased in volume showed that both had their pre-SRS MRI within 1 month of their initial post-op MRI. One patient in this group showed tumor progression within the resection cavity wall and had a 12% expansion of their cavity (2.6 cm^3^). The second patient had an 18% (1.2 cm^3^) expansion and showed no tumor progression on pre-SRS MRI.

Eleven patients (52%) had evidence of tumor progression within the resection cavity wall at the time of pre-SRS imaging. Five patients were in the <1 month group, and six patients were in the ≥1 month group. Interestingly, initial post-operative cavity volume and time between post-operative and pre-SRS images were not associated with tumor progression (p=0.28 and p=0.46, respectively). Additionally, each pathologic tumor type except ovarian carcinoma and sarcoma had evidence of tumor progression.

## Discussion

SRS is a promising adjuvant therapy after surgical resection of brain metastases. It has been shown to limit local progression at levels comparable to WBRT while potentially sparing patients the neurocognitive risks of WBRT [6-7, 12-20]. Since the risks associated with SRS, such as radionecrosis, occur in >10% of patients [21-23], smaller target volumes reduce the amount of radiation exposure to the surrounding normal brain. Limiting the target volume can therefore potentially reduce the risk of adverse affects associated with SRS. In this retrospective study, we show that brain metastasis resection cavities appear to achieve, on average, the greatest amount of constriction after 1 month. In addition, tumor progression, even in the setting of a gross total resection, is common in the interval between surgery and SRS. Interestingly, Smith et al., showed that early enhancement after resection of gliomas is often associated with diffusion abnormalities on MRI and thus may not necessarily reflect recurrent tumor but instead represent a response to the trauma of surgery [24].

Prior studies have shown that brain metastasis resection cavities undergo dynamic volume changes, although there are conflicting data as to the exact pattern of change. Jarvis et al., found that nearly 50% of resection cavities examined remained unchanged in size (defined as ≤2 cm^3^ change in volume), while 23% showed a reduction in volume, and 30% showed volume expansion [10]. The mean time between initial post-operative and pre-SRS scans was 24 days (range 2-104). Interestingly, those patients whose volumes expanded had a mean time between scans of 19 days. Atalar et al., reported a population of brain metastasis patients treated with surgical resection and adjuvant SRS with a median time between scans of approximately 20 days [11]. They also compared the pre-operative tumor volume to the initial post-resection cavity volume and concluded that the majority of volume change occurs before the initial post-resection scan (i.e., due to resection of the tumor) with minimal changes occurring in the interval between the initial post-resection and pre-SRS scans. However, the longest such interval in their series was only 33 days, so it is possible that further cavity reductions could have been detected had they waited longer before proceeding with adjuvant SRS. At least nine patients reported showed expansion of their resection cavity with three showing sizable changes (~50-200%). Similarly, in our series, close examination of individual patients reveals that all cases of cavity expansion occurred in patients whose pre-SRS MRI scan was performed within 1 month of surgical resection. No patients who had a pre-SRS scan performed after 1 month showed expansion. Review of medical records found no indication that patients with expanding resection cavities were scheduled for adjuvant SRS any earlier than other patients or that they developed symptoms related to mass effect. Additionally, since there was no significant relationship between change in cavity volume and surrounding edema, our results suggest that use of steroids will not affect the size of the resection cavity volume at the time of SRS. Our hypothesis before this review was that surrounding edema would increase local/regional tissue pressures and thereby promote compression of the cavity and a greater decrease in relative size than those without abundant edema. This was not the case.

Taken together, the data from the present study and priors can be interpreted in one of two ways. First, resection cavities may tend to constrict with time even if there is an initial expansion. Waiting more than 1 month to initiate SRS may then maximize the potential resection cavity constriction. Conversely, these data may suggest that while most resection cavities constrict over time or remain relatively unchanged, there is a distinct population that undergoes expansion. As suggested by Jarvis et al. [10], reasons for expansion may include cystic changes, tumor progression, and non-specific post-surgical changes. Early post-resection imaging may help to detect patients with expanding cavities in order detect tumor progression or begin SRS earlier or switch the modality of radiation treatment to a fractionated regimen.

Unfortunately, an important limitation of this study and others is that only two imaging time points – early post-resection and pre-SRS – were used to define resection cavity volume dynamics.  Such a technique assumes linearity of cavity volume dynamics. A better understanding of true cavity volume dynamics could be obtained by having more interval imaging studies for a given patient. This would be especially beneficial in those patients whose cavities show expansion as it would help determine if cavity expansion is a transient process followed by constriction, a linear process that may eventually lead to symptoms from mass effect or an asymptotic process. Additionally, we cannot exclude that a relationship exists between pathologic tumor type and rate of tumor expansion, but the small number of patients in each group is another limitation in this study.

The obvious risk of delaying adjuvant SRS is tumor progression in the interval between surgical resection and SRS. Jarvis et al., reported local progression in 8.6% of patients with a gross total resection and 37.5% with a subtotal resection. In our series, nearly half showed contrast enhancement on a pre-SRS planning MRI. Additionally, we did not observe a difference in pathology affecting recurrence rate. Tumor progression in the interval between surgery and adjuvant SRS may necessitate treatment to a larger volume or indicate a worse prognosis.

## Conclusions

These data confirm that dynamic changes occur in the volumes of post-resection cavities for brain metastases and that tumor progression is a common phenomenon, even in patients with a gross total resection. Specifically, our data suggest that the greatest cavity constriction is seen in patients whose pre-SRS imaging is ≥1 month after initial post-operative imaging. If cavities that initially expand may eventually constrict, earlier imaging may lead to unnecessarily early intervention. We, therefore, do not see compelling evidence to repeat imaging before the 1 month post-resection period. It is also important to acknowledge that this study only evaluates the dynamic changes in cavity volume and does not address the critical issue of how these changes or the timing of adjuvant SRS affect the outcome. Further studies will be required to answer this question.
